# A Review: Research Progress in Modification of Poly (Lactic Acid) by Lignin and Cellulose

**DOI:** 10.3390/polym13050776

**Published:** 2021-03-03

**Authors:** Sixiang Zhai, Qingying Liu, Yuelong Zhao, Hui Sun, Biao Yang, Yunxuan Weng

**Affiliations:** 1 College of Chemistry and Materials Engineering, Beijing Technology and Business University, Beijing 100048, China; 1803020140@st.btbu.edu.cn (S.Z.); 1803020133@st.btbu.edu.cn (Q.L.); 13552134315@163.com (Y.Z.); ybiao@th.btbu.edu.cn (B.Y.); 2 Beijing Key Laboratory of Quality Evaluation Technology for Hygiene and Safety of Plastics, Beijing Technology and Business University, Beijing 100048, China

**Keywords:** poly (lactic acid) (PLA), lignin, cellulose, nanocellulose, microcrystalline cellulose, composite materials, modification

## Abstract

With the depletion of petroleum energy, the possibility of prices of petroleum-based materials increasing, and increased environmental awareness, biodegradable materials as a kind of green alternative have attracted more and more research attention. In this context, poly (lactic acid) has shown a unique combination of properties such as nontoxicity, biodegradability, biocompatibility, and good workability. However, examples of its known drawbacks include poor tensile strength, low elongation at break, poor thermal properties, and low crystallization rate. Lignocellulosic materials such as lignin and cellulose have excellent biodegradability and mechanical properties. Compounding such biomass components with poly (lactic acid) is expected to prepare green composite materials with improved properties of poly (lactic acid). This paper is aimed at summarizing the research progress of modification of poly (lactic acid) with lignin and cellulose made in in recent years, with emphasis on effects of lignin and cellulose on mechanical properties, thermal stability and crystallinity on poly (lactic acid) composite materials. Development of poly (lactic acid) composite materials in this respect is forecasted.

## 1. Introduction

As an important family of materials, polymers are finding more and more applications. Most polymers are petroleum-based, with stable chemical properties and can persist for a long time in the natural environment. However, synthetic polymer production (300 Mt/year) and ordinary management practices have caused irretrievable harm to the environment [1]. In light of this, exploration of biodegradable polymers is expected to offer solutions to such problems. Worldwide bioplastics commercial growth is increasing and made up approximately 25–30% of the overall plastic market by year 2020. Considering the rising demand for energy and decline of fossil resources, the global economy is currently aiming to replace recognized energy sources by greener, bio-based, and sustainably produced equivalents [2,3]. Biodegradable polymers are considered to be promising alternatives. Examples of their applications are in cardiac blood tube support [4], medical instruments, agricultural mulch [5], food packages [6], disposable tableware [7], sutures [8], and so forth.

**Figure 1:**
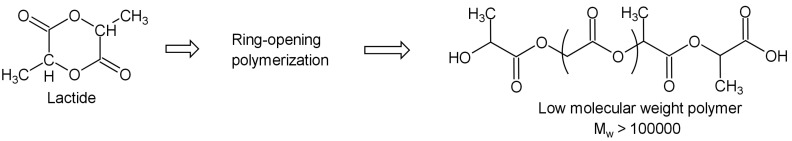
Ring-opening polymerization of lactide.

**Figure 2:**
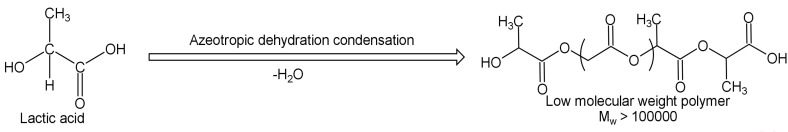
Direct condensation polymerization of lactic acid.

Biodegradable polymers can be degraded by microorganisms in nature and finally become harmless small molecular substances such as water and carbon dioxide, giving no rise to environmental issues when abandoned [9,10]. Biodegradable polymers can be divided into three categories—i.e., polymers from nature (biopolymers such as starch, protein, etc.), microbial synthesis (a representative of which is polyhydroxyalkanoates), and chemical synthesis (such as polyvinyl alcohol and polybutylene succinate, etc.). Typical biodegradable polymer poly (lactic acid) (PLA) belongs to the chemical synthesis class, which can be prepared by ring-opening polymerization of lactide (Figure 1) or direct condensation polymerization of lactic acid (Figure 2) [11]. PLA has the characteristics of excellent degradation [12], biocompatibility, nontoxicity [13], and good workability and thermoplasticity among others [14]. PLA can be conveniently processed into thin films, fibers or molding parts [15]. However, PLA has some shortcomings, such as low crystallization rate and poor impact properties and thermal resistance [16,17,18,19]. Modification of PLA to overcome such drawbacks has become a research area that attracts much interest [20].

A number of previous studies have shown that biological materials extracted from plants can be used in many ways to help early blight disease in solanum lycopersicum and antineoplastic potency [21,22]. Lignin and cellulose are the two of the most abundant organic resources in nature and can be obtained from various resources and have much greater potentials than the resources currently under development.

**Figure 3:**
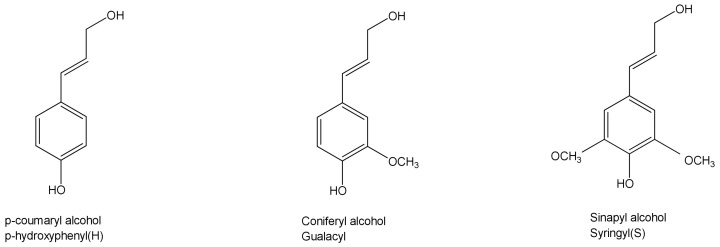
The structure of lignin.

Lignin, a kind of multiphase polymer of high molecular weight with a large amount of aromatic structures, has excellent thermal stability and good mechanical properties. According to the content of the methoxy group, the structure of lignin can be divided into three phenylpropane units (Figure 3), which are classified as guaiacyl propane unit (G-type lignin), syringyl propane unit (S-type lignin) and p-hydroxyphenyl propane unit (H-type lignin) [23]. Lignin can also be used as a raw material of aromatic compounds and benzene derivatives. In addition, it can be used as a burning material in a paper mill, synthetic adhesive or cement additive [24], showing great economic benefits.

Cellulose is a biological macromolecule formed by connecting D-pyranose glucose rings with β-1,4-glycosidic bonds. Due to its abundance in nature, being biodegradable, nontoxicity, good compatibility, and low price and such, cellulose has also been widely used in paper making, environmental protection, food processing, and other areas [25].

Lignin and cellulose have many excellent properties and rich contents. Blending lignin, cellulose, and PLA can improve the performance of materials to a certain extent for application in packaging, medicine, construction, and other fields. However, there are still problems to be solved in the process of blending. First, lignocellulosic materials contain a large number of hydrophilic polar carbonyls, while PLA contains a large number of hydrophobic nonpolar carbonyls, resulting in poor compatibility and interfacial adhesion. In the case of direct blending, the effect of lignocellulosic materials on the crystallization and mechanical properties of PLA is not obvious. To improve the compatibility of lignin, cellulose, and hydrophobic poly (lactic acid), surface modification of lignin and cellulose can be carried out, such as acetylation, silylation, grafting, and use of coupling agents. Second, lignin, cellulose, and hemicellulose make up the main components of lignocellulosic materials [26]. There is a strong physical and chemical interaction between the three. The combination of them must be destroyed by pretreatment, so as to separate a single pure component [27,28,29]. Therefore, the cost of pretreatment has become a point that must be considered for commercial purposes.

This paper mainly reviews the research progress in the modification of poly (lactic acid) composites by lignin, nanocellulose, and microcrystalline cellulose, with emphasis on the effects of lignocellulosic materials on the mechanical properties, crystallinity, and thermal stability of poly (lactic acid).

## 2. Lignin/PLA Composite Materials

A wealth of the current literature has reported modifications of PLA with lignin, which can be divided into lignin modified PLA and modified lignin modified PLA.

### 2.1. Lignin Modified PLA

Zhou et al. [30] studied the influence of the addition of lignin on the mechanical properties and crystallinity of poly (lactic acid). The results showed that the tensile strength and elongation at break of composite materials decrease, while the elastic modulus increases. At a 20% lignin loading, the composite materials have tensile strengths of 31.9% lower than that of pure PLA and elongations at break that are 74.5% lower, but elastic moduli that are increased by 10.21%. This may be the peaks of hydroxyl groups of lignin and carbonyl groups of PLA and the hydrogen bonding interactions between them [31]. Another possible explanation for this observation is that the addition of lignin will hinder the long-range continuous formation of PLA molecules, leading to a decrease in the tensile strengths and elongations at break of PLA/lignin composites. In a separate study, Ma et al. [32] blended PLA with various lignin additions (50, 60, and 70%), and found that the changes of tensile strength and elongation at break of the composites also showed similar trends (Table 1). The elastic modulus was also found to increase, but not all the way with the increase in lignin content.

In addition to the effect of lignin on mechanical properties of PLA/lignin composites, influence of lignin on crystallization of PLA has also been explored [30,31]. Zhou [30] and Singla [31] investigated how lignin addition affects the crystallinity of PLA/lignin composites, respectively. It was found that molecular weight of lignin has a great influence on crystallization nucleation of PLA. The addition of lignin of low molecular weight will make the cold crystallization temperature (*T*_cc_) of the material increase first and then decrease, so the crystallinity tends to decrease at first and then increase; at a 20% lignin content, the degree of crystallization exceeds that of pure PLA, reaching 10.48%. The reason for this may be that lignin with a low molecular weight acts as a crystallization nucleating agent, resulting in an increase in the degree of crystallinity. When the content of lignin is low, the movement of the PLA molecular segment is limited by the rigid group in lignin, giving rise to decreased crystallinity. However, with augmented lignin content, the effect of nucleation becomes stronger, and the crystallinity of poly (lactic acid) can be enhanced [33]. In contrast, addition of lignin of high molecular weight was found to elevate the *T*_cc_ and reduce the degree of crystallization. The reason for this observation may be that the changes in the cold crystalline peaks of PLA in the composites can be explained on the basis that, during the heating process, lignin becomes a highly viscous tarry product. High viscosity lignin will affect the movement of PLA chain segments and hinder their crystallization [31].

The addition of lignin also affects the thermal stability of PLA. Due to the lower onset thermal degradation temperature (*T*_onset_) and maximum thermal degradation temperature (*T*_max_) of lignin compared to PLA, lignin will decompose prior to PLA, leading to decreased *T*_onset_ and *T*_max_ of PLA/lignin composites; reductions in the degradation temperatures were found to be more drastic with the augmentation of lignin addition [33] (Table 2).

Crosslinking, chemical or physical, is a process in which linear or branched polymer chains are covalently linked to form a network or shaped polymer. Chemical crosslinking is usually realized by polycondensation and addition polymerization, such as vulcanization of rubber and curing of unsaturated polyester resin; physical crosslinking uses light and heat radiation to crosslink linear polymers. After moderate crosslinking, the mechanical strength, elasticity, dimensional stability and solvent resistance of linear polymers are improved. Crosslinking is thus commonly used in modification of polymers.

**Figure 4:**
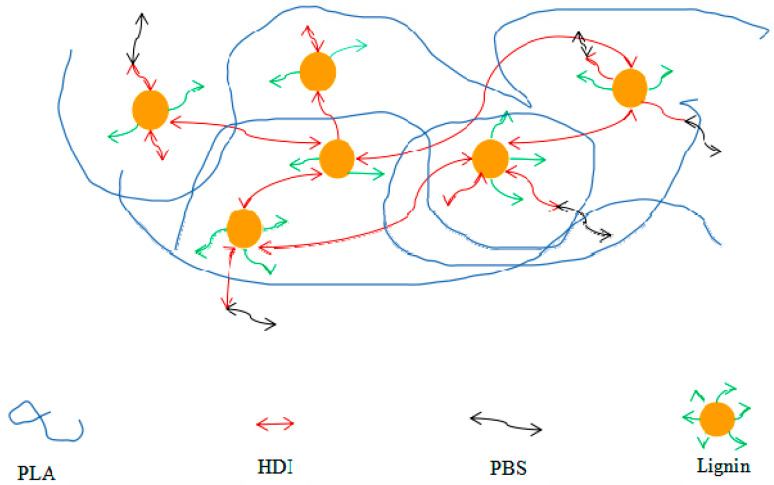
The semi-interpenetrating network of poly (lactic acid) and lignin.

A three-dimensional network polymer can be obtained by interconnecting the linear polymer chains with chemical bonds. Liang [34] compounded poly (butylene sebacate) (PBS) with PLA and used hexamethylene diisocyanate (HDI) to connect PBS with hydrolyzed lignin, thus constructing a semi-interpenetrating network of PLA and lignin (Figure 4). It was found that a suitable amount of PBS and lignin can raise the tensile strength and elongation at break of the composites. At low PBS contents, increasing lignin addition can effectively lift the tensile strength. However, at high PBS contents, the addition of lignin and PBS is not conducive to elevating the strength of the composites because of both the flexibility of PBS itself and the incompatibility between PBS and lignin. In addition, the elongation at break is also affected by the compatibility between PBS and lignin. At fixed PBS contents, the elongation at break will rise with the decrease in lignin content. Therefore, by controlling PBS and lignin contents, the strength and elongation at break of PLA composites can be tuned.

Though mixing lignin with PLA could not help improve the thermal properties and crystallinity of PLA, addition of lignin was found to lift the overall stiffness and modulus of the composites, which is favorable with regard to its use certain fields—i.e., food packaging.

### 2.2. Modified Lignin Modified PLA

Although the structure of lignin from different sources may differ slightly, it generally contains active functional groups such as aryl, hydroxyl, methoxy and double bonds. These functionalities provide reaction sites to offer various possibilities for chemical modification of lignin via esterification, sulfonation, amination or graft copolymerization [35,36]. Lignin can be consumed by most of the hydroxyl groups on the surface through chemical modification, thus reducing hydrogen bonding interaction with PLA and improving compatibility between lignin and PLA. Therefore, the physical and chemical properties of lignin/PLA composite materials can be improved. Research shows that compared with acidified lignin composites and butyric lignin composites, acetoacetate lignin composites have better compatibility, thermal stability, and mechanical properties due to hydrogen bonding interactions [37].

Subsequent composite of modified lignin with PLA is expected to further improve the mechanical properties and thermal properties of composites for a broader application.

#### 2.2.1. Esterification

Via esterification, alcohols react with carboxylic acids or carboxylic acid derivatives (acid chlorides or anhydride) to form esters. Vila [38] esterified butyric anhydride, isobutyl butyric anhydride, and crotonic anhydride, respectively, with lignin, to study the effect of these esterified lignins on the mechanical properties of PLA. It was found that the butyric anhydride and isobutyl anhydride modified lignins can bring the Young’s moduli of the composites to a level close to that of pure PLA, and significantly improve the fracture strain and plasticity of PLA. However, lignin modified by crotonic anhydride showed no improvement in plasticity.

In addition to types, the amount of esterified lignin loading also affects the mechanical properties of PLA. 

Liang et al. [39] studied the effect of maleic anhydride modified lignin (Lignin-Ma, LM) on the properties of PLA/epoxidized soybean oil (ESO). It has been found that LM and ESO can improve the crystallinity of PLA. The reasons are twofold: one is that LM and ESO act as nucleating agents, promoting the crystallization of PLA; the other is that ESO as a plasticizer can improve the flexibility of the PLA chain, so that the PLA chain can be sequentially arranged into crystal structure at low temperatures. The addition of LM also improved the thermal stability of PLA/ESO composites, with the *T*_onset_ of the composite reaching 300 °C. 

For composites with different ESO loadings, i.e., 10 vs. 20%, the influence of LM on the mechanical properties is completely different. When 10% ESO is added, low content of LM enhanced the toughness because LM reacted with ESO and weakened the incompatibility between ESO and PLA. At 20% ESO addition, low LM loading significantly enhanced the mechanical properties as well. Further increasing LM content would weaken the mechanical properties of PLA/ESO/LM composites because excessive LM would agglomerate rather than react with ESO.

Analogously, Guo et al. [40] studied succinylated lignin (SAL) obtained by succinic anhydride (SA) modified lignin on the properties of PLA/ESO composites. 

SAL was found to elevate the Young’s moduli of PLA/SAL/ESO composites. The composites have Young’s moduli higher than those of both pure PLA and PLA/ESO. This reveals that the addition of ESO improves the dispersion of PLA and interfacial interactions with PLA, indicating a positive impact on the miscibility of PLA. Compared with pure PLA, the composites had smaller particle sizes and smoother surfaces. This was attributed to the reactive compatibilization between the carboxyl of SAL and epoxy groups of ESO, which improves the properties of PLA/SAL/ESO composites by dynamic vulcanization. At a 4 % ESO volume, PLA/SAL/ESO composites showed relatively homogeneous morphologies with much smoother surfaces, demonstrating a superior interfacial interaction.

#### 2.2.2. Sulfonation 

The introduction of sulfonic acid groups into lignin was found to effectively improve the water solubility of lignin, rendering it hydrophilic. Sulfonated lignin can be used in various fields. For example, the thermal stability of urea-formaldehyde resin can be improved by adding sulfonated kraft lignin. Sulfonated kraft lignin addition also helps reduce the amount of formaldehyde release, significantly improving the performance of adhesive [41]; lignosulfonate/PLA composites can be better used in 3D printing technology or public products [42].

Hu et al. [43] found that lignosulfonate ammonium (OMAL) can improve the mechanical properties and crystallinity of PLA/wood fiber (PLA/WF). OMAL-PLA/WF showed the best mechanical properties at 15~20 wt% OMAL addition. The enhancement in mechanical properties by OMAL can be explained by two reasons: a major one is the activity of OMAL which promotes the movement of the PLA chain segment, leading to the entanglement and crosslinking of PLA with wood fiber and lignin ammonium sulfonate; the minor one is associated with the adhesion ability of lignin ammonium sulfonate.

#### 2.2.3. Silanization

The introduction of silane groups, normally for the purpose of replacing active hydrogen, can be used to reduce the polarity and thus hydrogen bond binding of the compound. 

**Figure 5:**
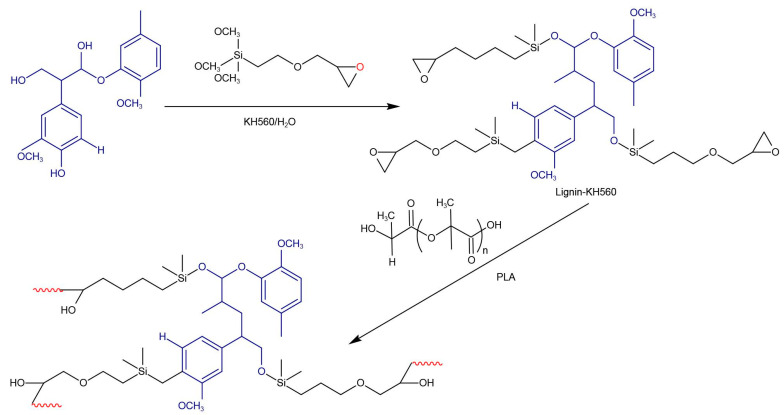
Reaction mechanism of coupling agent γ-(2,3-epoxypropoxy) propyl trimethoxysilane (KH560) and PLA/lignin composite.

Wang et al. [44] prepared PLA/lignin composites via a one-step solvent-free modification method (Figure 5) using or without using the silane coupling agent γ-(2,3-epoxypropoxy) propyl trimethoxysilane (KH560) and studied its effects as a compatibilizer on the mechanical properties and crystallization behavior of a PLA/lignin composite.

It was found that the coupling agent (KH560) increased the interfacial adhesion between PLA and lignin. The tensile strengths of the modified composites were elevated from 45.7 (5%LG/PLA) to 50 MPa (5% LG-KH560/PLA), indicating the enhancement of mechanical properties of the composites by the coupling agent.

In the case of 1%LG/PLA and 3%LG/PLA, the cold crystallization peak temperature changed only slightly. The reason for this phenomenon could be the increase in the surface area of heterogeneous nucleation, which is beneficial to the crystal formation under colder conditions, leading to the increasing trend of crystal formation. In the low temperature crystallization process of 5%LG/PLA, the peak temperature of cold crystallization shifted to a high temperature, which is different from the low temperature crystallization phenomenon. This phenomenon is due to the fact that the resistance to the movement of the molecular chains required for crystallization increases more than the rise in crystal formation rate due to heterogeneous nucleation. The *T*_cc_ of the PLA/lignin composites using KH560 was lower than that without using KH560. After the modification of KH560, *T*_cc_ of the three-component composites decreased. The reason for this may be that the long linear aliphatic side chain of KH560 can act as heterogeneous nucleating agent, resulting in a decrease in crystallization potential. Compared with the KH560 modified composite, the unmodified lignin has a poor binding property to PLA, which led to steric hindrance restricting the movement of PLA molecular chains. In addition, it limited the crystallization of PLA, causing an increase in glass transition temperature (*T*_g_) and *T*_cc_. 

Compared with direct blending, though modified lignin addition could not improve the thermal stability of PLA, this approach can further improve the mechanical properties and crystallinity of PLA/lignin. Different chemical modification methods and modified lignin addition can be used in tailoring the properties for various purposes. Chemical modification of lignin needs to be further studied so that PLA/lignin composites can expand the scope of application.

## 3. Cellulose/PLA Composite Materials

### 3.1. Nanocellulose

As a new type of green nanomaterial, nanocellulose has received extensive attention in the energy storage area in recent years. In addition to the natural advantages of abundant storage and cyclic regeneration, nanocellulose also has strengths such as a superior surface area and good mechanical properties [45,46], and thus can be used in medical treatment, packaging, food industry and other fields [47]. Nanocellulose is the general name of cellulose nanomaterials. This review mainly covers modification of PLA by nanocellulose (NC) and nanocrystalline cellulose (NCC).

#### 3.1.1. Nanocellulose Modified PLA

Alana et al. [48] extracted nanocellulose (NC) from cotton waste and industrial waste and modified PLA with these two kinds of nanocellulose. It was found that both cotton waste-nanocellulose (CW-N) and industrial waste-nanocellulose (IW-N) improved the toughness of PLA due to the spatial arrangement of the fibers. At the same time, the addition of CW-N and IW-N proved to improve the flexibility and elongation at break of the composite as well.

Huang et al. [49] prepared nanocellulose using cassava residue as raw material and blended different contents of nanocellulose with PLA for composite preparation. The results showed that the tensile strength of PLA/nanocellulose composite films increased when less than 1.0% of nanocellulose was added. At nanocellulose addition higher than 1.0%, NC produced microaggregates by itself, and was difficult to evenly disperse in PLA, resulting in a decline in tensile strength. In addition, compared with NC with 1.0% or higher lignin, adding 0.5% NC was found to improve the flexibility of the thin film, because most of the active groups of nanocellulose in the small amount of nanocellulose were absorbed by the PLA chain. The dispersion stress not only increased the tensile strength of the material, but also reduced the bonding strength between the PLA molecules, improving the overall flexibility of the thin films. When adding 1.0% nanocellulose, the nanocellulose formed a stable network structure with PLA through entanglement and cocrystallization of the molecular chain, which enhanced the forces between molecules. This enhanced the tensile strength and increased the elastic modulus. When part of the network chain breaks due to the “cross-linking” role of nanocellulose, the other molecular chains in PLA can still withstand stress.

Preparation methods were also found to affect the properties of composites. Sullivan [50] prepared PLA/NCC composite films by melt mixing and compression molding. This showed that bionanocomposites films have more surface fracture events as compared to pure PLA, which indicated that the incorporation of NCC contributed to a more brittle PLA. By increasing the amount of NCC, the crystallinity of the thin films was further improved by the crystallization with NCC. Generally, NCC played the role of a nucleating agent, which increased the crystallinity of the polymer matrix and improved the crystallinity and toughness.

Zhang et al. [51] studied the mechanical, rheological, and thermal properties of PLA/nanocellulose bionanocomposite films fabricated by using Pickering emulsion. This showed that NCC loading improved the transition of films from liquid to solid-like state at high temperature. The onset crystallization temperature increased, indicating that the addition of NCC as a nucleating agent promoted the crystallinity of the polymer. The onset temperature of thermal decomposition also increased as NCC was added.

#### 3.1.2. Modified Nanocellulose Modified PLA 

Chemical modification can be used to tailor the properties of materials at the molecular level. With esterification and grafting modification of nanocellulose, its composites with PLA show significantly enhanced mechanical properties and thermal stability, and thus can be applied in the fields of packaging, medical treatment, agriculture and so on.

##### Esterification

Liu et al. [52] prepared nanocomposites by compounding PLA and acetate nanocellulose (ANC), which was obtained by acetalization and hydrophobic modification. It was found that ANC showed no significant effect on the mechanical properties of PLA. The addition of ANC can also reduce the elongation at break of composite films while increasing the yield strength. However, acetate nanocellulose was found to significantly improve the crystallization degree and crystallization rate of PLA (Table 3). The reason for this phenomenon could be that ANC played a role as heterogeneous nucleating agent for PLA crystallization. Moreover, it was also found that the yield strength and elongation at break of pure PLA films prepared in chloroform and N, N- dimethylformamide (DMF) are different, because PLA is a crystalline polymer and the preparation conditions of the films affect the mechanical properties of PLA mainly by affecting its crystallization properties.

In addition to the mechanical properties and crystallization enhancement by ANC, acetylated nanocrystalline nanocellulose (ANCC) was found to greatly improve the properties of PLA as well.

Ling et al. [53] prepared ANCC using sulfuric acid followed by acetylation. The effect of ANCC content on PLA/ANCC properties was studied. Results showed that adding an appropriate amount of ANCC increased the tensile strength of the PLA/ANCC composite, mainly because the added ANCC was uniformly dispersed in the PLA matrix, playing a role in strengthening fiber dimension. However, if excessive ANCC was added, the tensile strength of the composite would decrease to some extent, which was due to the agglomeration phenomenon of ANCC. The uneven distribution in composites disrupted the original uniform PLA matrix, resulting in stress concentrations in some parts and thus weakening mechanical properties. 

Similarly, Ming et al. [54] studied the influence of different contents of acetylated nanocrystalline cellulose (ANCC) on the properties of PLA/ANCC composites. The results showed that the tensile strength and Young’s moduli of composites first rose and then declined with augmenting ANCC content. When ANCC content was 8%, the tensile strength was higher than that of pure PLA; at higher ANCC contents, the tensile strength showed a downward trend. For example, at 12% ANCC content, the tensile modulus was reduced to 26.1 MPa. An explanation for this change could be the worsening dispersion of ANCC in the PLA matrix with more ANCC addition. In the local stress concentration caused by aggregation of ANCC, tensile failure occurred too early in the testing process. Overall, at 8% ANCC addition, the tensile strength of PLA/ANCC composites reached the maximum.

##### Graft Modification

In grafting modification, certain functionality was used to replace the hydrogen of the hydroxyl groups of nanocrystalline cellulose. This graft modification helped prevent the self-aggregation of nanocrystalline cellulose and also kept the structure and properties of nanocrystalline cellulose basically unchanged. 

Wang [55] prepared esterified nanocrystalline cellulose using maleic anhydride first. Then, methyl acrylate (MA) and butyl acrylate (BA) was used in graft modification of the nanocrystalline cellulose that underwent esterification. The thus modified nanocrystalline cellulose (ENCC-g)/PLA composite was studied with respect to its mechanical properties. It was found that the tensile strengths and elastic moduli of PLA composites increased in the initial stage but decreased afterwards with increasing ENCC-g content. At 2% ENCC-g loading, the tensile strength and elastic modulus reached the maximum—i.e., 56.82 and 1428.6 MPa, respectively. The reason for this variation could be that the hydrophobic group grafted on ENCC-g improved the compatibility between nanocrystalline cellulose and the PLA matrix, which accounted for the improvement in mechanical properties of the composites. However, at higher loadings, ENCC-g would partially agglomerate and reduce the interface compatibility, leading to reduced mechanical properties. Additionally, ENCC-g showed better interface compatibility with PLA and could disperse in PLA more evenly, giving rise to stronger nucleation effect of NCC, and therefore significantly improving the crystallinity of the composites.

Compared with the modification of PLA using unmodified nanocellulose or nanocrystalline cellulose, modification with modified nanocellulose or nanocrystalline cellulose could improve both mechanical properties and the crystallization degree/crystallization rate.

### 3.2. Microcrystalline Cellulose

Microcrystalline cellulose (MCC) is a refined form of wood pulp and a natural fiber with the highest specific surface [56]. MCC can be used as drug shaping agents and tablet disintegrating agents in the medical and pharmaceutical industries; MCC can also be used as thickening agents and emulsifying agents for water-based coatings due to its thixotropy and thickening properties; MCC combines filling, thickening, and emulsifying and has a good emulsifying ability for oily substances. Owing to its characteristics of complete biodegradation, strong rigidity, and high crystallinity, MCC can be used as a reinforcing agent to improve the mechanical properties and thermal stability of polymers [57]. MCC was also found effective in strengthening PLA.

#### 3.2.1. Microcrystalline Cellulose Modified PLA

In blending modification, the structure and microscopic composition in the microcrystalline cellulose system were modified with the help of nonchemical bonds, leading to the improvement of macroscopic mechanical properties such as tensile strength and elongation at break.

Xian et al. [58] studied the influence of different contents of MCC on the mechanical properties of PLA/MCC composites. The results showed that with the increase in MCC content, the tensile strength of composites increased at first and then decreased. MCC content of 4 wt% gave the highest tensile strength—i.e., 73.01 MPa, which is 8.40% higher than that of pure PLA. The tensile modulus also increased from 206 to 262.9 MPa. Enhancement of mechanical properties by MCC can be explained by its larger surface area, great activity, and hydrogen bonds formed between PLA and MCC, which contributed to the uniform dispersion of MCC in PLA matrix and good combination with its boundary surface.

#### 3.2.2. Modified Microcrystalline Cellulose Modified PLA

Through different chemical modifications such as sulfonation and grafting, microcrystalline cellulose can be modified with respect to its thermal stability, mechanical properties, crystallinity, and such. The modified microcrystalline cellulose could be added to PLA for various applications such as food packaging, liquid containers, plastic bags, disposable cups, etc. [59,60,61,62].

##### Sulfonation

Zhao et al. [63] prepared sulfonated microcrystalline cellulose (S-MCC) using sulfuric acid for subsequent preparation of S-MCC/PLA composites by a solvent casting method. In an analogous manner, the change in tensile strength of S-MCC/PLA composites followed an inverted V type trend with increasing S-MCC addition. At 9% MCC mass fraction, the S-MCC/PLA composite showed a tensile strength of 1671 MPa, much higher than 1181 MPa for MCC/PLA, indicating a remarkable enhancing effect by sulfonation. This effect could be due to the fact that a large number of hydroxyl groups were hydrolyzed on the surface of MCC, which contributed to the improved miscibility of MCC. A separate study on thermal gravimetric analysis of MCC/PLA composites by Frone et al. [64] found that its thermal stability was obviously improved. More specifically, unmodified MCC/PLA composites underwent thermal decomposition between 260 and 280 °C, while S-MCC/PLA began to decompose at 300 °C. Therefore, the sulfonation modification of MCC made its composite with PLA more thermally stable, with an even higher decomposition temperature of 300 °C.

##### Graft Modification

Grafting polymerization, especially “grafting from” free radical polymerization, was used to modify the surface properties of cellulose fibers, generally through carbamate or interlayer adsorption. For example, a facile method to graft biodegradable starch on fiber surface through the hydrogen bond formations among cellulose, starch, and ammonium zirconium carbonate was developed [65]. 

**Figure 6:**
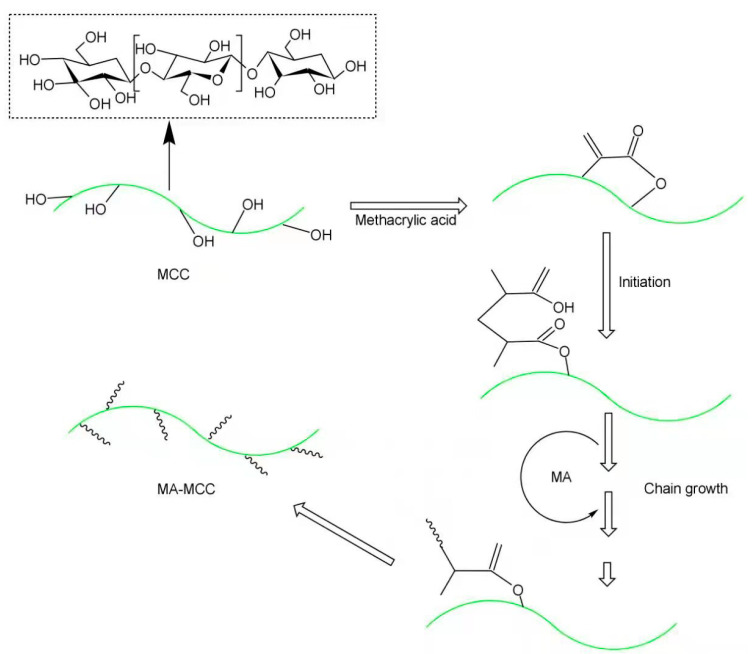
Surface modification of microcrystalline cellulose.

Zhu et al. [66] obtained methacrylic acid grafted MCC (MA-MCC) by a grafting polymerization method [67] (Figure 6) for preparation of an MA-MCC/PLA composite. It was found that MA-MCC had better compatibility with and better dispersion in the PLA matrix and further increased the tensile strength of PLA composite by 52.6 MPa. At the same time, after adding MA-MCC, the maximum impact strength of the composite reached 8.16 kJ/m^2^. In addition to the enhancement of mechanical properties, MA-MCC also affected the *T*_cc_ of PLA. The cold crystallization temperature of PLA dropped to only 116.8 ℃. MA-MCC could be used as a nucleating agent [68], which weakened the clustering phenomenon of PLA and showed better dispersion in PLA. In the crystallization process, MA-MCC helped increase the fluidity of the PLA chain, making it easier to crystallize, resulting in a lower cold crystallization temperature. In contrast, unmodified MCC is prone to caking, and is too large to break the PLA chains so as to form nucleation points. Therefore, the modified MCC served to improve the mechanical properties and reduce the crystallization temperature as well.

## 4. Challenges and Commercial Value

Compared with lignin and cellulose, modified lignin and cellulose can improve the performance of PLA more prominently. Owing to its excellent performance, PLA can be used in many fields, such as food packaging, medical devices, agriculture, etc. Furthermore, for PLA composite materials, their mechanical properties, thermal stability, and crystallinity have changed accordingly, making them more promising in various fields (Table 4).

Due to reasons including a lower energy content than coal, lignin has a limited energy value [69]. As such, valorization of lignin is expected to enhance operational efficiency. In this regard, wise utilization of lignin for green biocomposites by combining with PLA to produce degradable materials with low environmental impact is surely of great significance and contributes to the creation of globally sustainable products [70]. In the future, the modification of PLA by cellulose and lignin is looking to have has good economic value and commercial prospect due to the green natures of both components. However, compared with other biodegradable biomass materials, there are still some shortcomings, which need to be improved. Further research also should be carried out on pretreatment methods to reduce the cost of manufacturing for a wider application. In the future, better modification methods can be found to further improve its performance.

## 5. Conclusions

As an environmentally friendly material, poly (lactic acid) (PLA) not only has good workability, but also good biocompatibility and degradability. However, examples of its shortcomings associated with practical application are less adequate mechanical properties and poor crystallization behavior. In order to overcome these shortcomings, various modification methods were conducted. This paper reviewed the effects of main biomass components, i.e., lignin, nanocellulose and microcrystalline cellulose, on PLA properties. Though mechanical properties and thermal stability of PLA/biomass composites were not always significantly improved by addition of lignocellulosic materials and thought certain properties may even be deteriorated, addition of modified lignocellulosic materials (modified ligin, nanocellulose/microcrystalline cellulose) was found to significantly enhance properties of PLA, including mechanical, thermal, and crystallization behaviors. 

Nowadays, PLA plays an important role in various fields such as transportation, food, agriculture, and medicine. Modification of PLA by blending with renewable and biodegradable lignocellulosic materials, either to enhance properties or to reduce cost to a certain degree, holds great potential, though there is still some step gap for improvement compared with chemical modification. Chemical modification of PLA through a molecular engineering approach will eventually prevail. It is believed wholly green PLA/lignocellulosic materials will be used in more applications eventually.

## Figures and Tables

**Table 1:** Performance parameters of poly (lactic acid) PLA/lignin.

Sample	Tensile Strength (MPa)	Elongation at Break (%)	Elastic Modulus (GPa)	References
Pure PLA	64.21	7.01	2.84	[30]
PLA/20%Lignin	43.71	1.79	3.13	[30]
Pure PLA	56	10.4	0.4462	[30]
PLA/50% Lignin	53.3	9.1	0.5221	[32]
PLA/60%Lignin	47.7	7.1	0.5161	[32]
PLA/70%Lignin	51.8	8.3	0.4695	[32]

**Table 2:** The degradation temperature of PLA/lignin.

Sample	Onset Thermal Degradation Temperature (*T*_onset_)/°C	Maximum Thermal Degradation Temperature (*T*_max_/°C)
Pure Lignin	266	346
Pure PLA	328	359
PLA/5%Lignin	326	351
PLA/10%Lignin	324	346
PLA/20%Lignin	319	345
PLA/30%Lignin	315	337

**Table 3:** Thermal and mechanical properties of PLA/acetate nanocellulose (ANC).

Sample	Yield Strength/MPa	Crystallinity (X_c_)/%	Crystallinity Time/min
PLA	36.5 ± 0.3	13	14.2
PLA/1.0%ANC	38.0 ± 0.5	16.3	8
PLA/1.5%ANC	43.1 ± 0.8	21.2	7.2

**Table 4:** The applications of pure PLA and PLA composite materials.

Sample	Applications
Pure PLA	Medical devices, Food packaging, Agriculture
Lignin/PLA	Food packaging, Sutures
Modified lignin/PLA	3D printing
Cellulose/PLA	Medical devices, Packaging
Modified cellulose/PLA	Plastic bags, Disposable cups, Liquid stuff container

## Data Availability

Data sharing not applicable.

## References

[1] Sirohi R., Pandey J.P., Tarafdar A., Sindhu R., Parameswaran B., Pandey A. (2020). Applications of poly-3-hydroxybutyrate based composite in advanced applications of polysaccharides and their composites. Mater. Res. Found..

[2] Bhatia S.K., Gurav R., Choi T.R., Jung H.R., Yang S.Y., Moon Y.M., Song H.S., Jeon J.M., Choi K.Y., Yang Y.H. (2019). Bioconversion of plant biomass hydrolysate into bioplastic (polyhydroxyalkanoates) using Ralstonia eutropha 5119. Bioresour. Technol..

[3] Venkata Mohan S., Nikhil G.N., Chiranjeevi P., Nagendranatha Reddy C., Rohit M.V., Kumar A.N., Sarkar O. (2016). Waste biorefinery models towards sustainable circular bioeconomy: Critical review and future perspectives. Bioresour. Technol..

[4] Li D.H., Zhen L., Yu P., Muhammadlqbal S., Yu F.Z., Li L. (2016). A review on biodegradable materials for cardiovascular stent application. Front. Mater. Sci..

[5] Serrano R.H., Martin C.L., Pelacho A.M. (2021). Biodegradable plastic mulches: Impact on the agricultural biotic environment. Sci. Total Environ..

[6] Mangaraj S., Yadav A., Bal L.M., Dash S.K., Mahanti N.K. (2019). Application of biodegradable polymers in food packaging industry: A comprehensive review. J. Packag. Technol. Res..

[7] Siakeng R., Jawaid M., Ariffin H., Sapuan S.M., Asim M., Saba N. (2019). Natural fiber reinforced polylactic acid composites: A review. Polym. Compos..

[8] Maharana T., Mohanty B., Negi Y.S. (2009). Melt–solid polycondensation of lactic acid and its biodegradability. Polym. Sci..

[9] Hamad K., Kaseem M., Ko Y.G., Deril F. (2014). Biodegradable polymer blends and composites: An overview. Polym. Sci. Ser. A.

[10] Somerville C., Youngs H., Taylor C., Davis S.C., Long S.P. (2010). Feedstocks for lignocellulosic biofuels. Science.

[11] Avérous L., Belgacem M., Gandini A. (2008). Polylactic acid: Synthesis, properties and applications. Monomers, Polymers and Composites from Renewable Resources.

[12] Cheng M., Qin Z.Y., Chen Y.Y., Hu S., Ren Z.C., Zhu M.F. (2017). Efficient extraction of cellulose nanocrystals through hydro-chloric acid hydrolysis catalyzed by inorganic chlorides under hydrothermal conditions. ACS Sustain. Chem. Eng..

[13] Wu Y.L., Wang H., Qiu Y.K., Loh X.J. (2016). PLA-based thermogel for the sustained delivery of chemotherapeutics in a mouse model of hepatocellular carcinoma. RSC Adv..

[14] Tripathi N., Katiyar V. (2017). Poly(lactic acid)/modified gum arabic based bionanocomposite films: Thermal degradation kinetics. Polym. Eng. Sci..

[15] Farah S., Anderson D.G., Langer R. (2016). Physical and mechanical properties of PLA, and their functions in widespread applications: A comprehensive review. Adv. Drug Deliv. Rev..

[16] Haafiz M.K.M., Hassan A., Khalil H.P.S.A., Fazita M.R.N., Islam M.S., Inuwa I.M., Marliana M.M., Hussin M.H. (2018). Exploring the effect of cellulose nanowhiskers isolated from oil palm biomass on polylactic acid properties. Int. J. Biol. Macromol..

[17] Shaheen T.I., Emam H.E. (2018). Sono-chemical synthesis of cellulose nanocrystals from wood sawdust using acid hydrolysis. Int. J. Biol. Macromol..

[18] Wang Z.H., Yao Z.J., Zhou J.T., Zhang Y. (2017). Reuse of waste cotton cloth for the extraction of cellulose nanocrystals. Carbohydr. Polym..

[19] Smyth M., Garcia A., Rader C., Foster E.J., Bras J. (2017). Extraction and process analysis of high aspect ratio cellulose nanocrystals from corn (*Zea mays*) agricultural residue. Ind. Crops Prod..

[20] Gottermann S., Standau T., Weinmann S., Altstadt V., Bonten C. (2017). Effect of chemical modification on the thermal and rheological properties of polylactide. Polym. Eng. Sci..

[21] Saratale R.G., Saratale G.D., Ghodake G., Cho S.K., Kadam A., Kumar G., Jeon B.H., Pant D., Bhatnagar A., Shin H.S. (2019). Wheat straw extracted lignin in silver nanoparticles synthesis: Expanding its prophecy towards antineoplastic potency and hydrogen peroxid sensig ability. J. Biol. Macromol..

[22] Pandey S., Giri V.P., Tripathi A., Kumari M., Narayan S., Bhattacharya A., Srivastava S., Mishra A. (2021). Early blight disease management by herbal nanoemulsion in solanum lycopersicum with bio-protective manner. Bioresour. Technol..

[23] Figueiredo P., Lintinen K., Hirvonen J.T., Kostiainen M.A., Santos H.A. (2018). Properties and chemical modifications of lignin: Towards lignin-based nanomaterials for biomedical applications. Prog. Mater. Sci..

[24] Gosselink R.J.A., Teunissen W., Van Dam J.E.F., de Jong E., Gellerstedt G., Scott E.L., Sanders J.P.M. (2012). Lignin depolymerisation in supercritical carbon dioxide/acetone/water fluid for the production of aromatic chemicals. Bioresour. Technol..

[25] Trache D., Hussin M.H., Chuin C.T.H., Sabar S., Fazita M.N., Taiwo O.F., Hassan T., Haafiz M.M. (2016). Microcrystalline cellulose: Isolation, characterization and bio-composites application-a review. Int. J. Biol. Macromol..

[26] Rinaldi R., Jastrzebski R., Clough M.T., Ralph J., Kennema M., Bruijnincx P.C.A., Weckhuysen B.M. (2016). Paving the way for lignin valorisation: Recent advances in bioengineering, biorefining and catalysis. Angew. Chem. Int. Ed..

[27] Guo J., Cao R., Huang K., Xu Y. (2020). Comparison of selective acidolysis of xylan and enzymatic hydrolysability of cellulose in various lignocellulosic materials by a novel xylonic acid catalysis method. Bioresour. Technol..

[28] Ufodike C.O., Eze V.O., Ahmed M.F., Oluwalowo A., Park J.G., Okoli O.I., Wang H. (2020). Evaluation of the inter-particle interference of cellulose and lignin in lignocellulosic materials. Int. J. Biol. Macromol..

[29] Sun W., Othman M.Z. (2019). A selective fractionation method of lignocellulosic materials using electro-assisted organosolv pretreatment. Bioresour. Technol..

[30] Zhou Z.P., Zhang W.W., Li Q., Yang G.S., Zhang H.H., Shao H.L. (2017). Structure and properties of lignin/polylactic acid composites. Polym. Mater. Sci. Eng..

[31] Singla R.K., Maiti S.N., Ghosh A.K. (2016). Crystallization, morphological, and mechanical response of poly(lactic acid)/lignin-based biodegradable composites. Polym. -Plast. Technol. Eng..

[32] Ma A.L., Xiao X.Y., Huang D.M., Zhang J.T. (2018). Study on mechanical properties of lignin/PLA composites. Guangdong Chem.Eng..

[33] Mu C.Y. (2014). Explorations for Processing and Properties of Lignin/Poly(*l*-Lactic) Acid Composites. Master’s Thesis.

[34] Liang X.L. (2019). Studies on Preparation and Properties of Poly(Lactic Acid)/Lignin Composites. Master’s Thesis.

[35] Zhang J.B., Ge Y.Y., Qin L., Huang W.X., Li Z.L. (2018). Synthesis of a lignin-based surfactant through amination, sulfonation, and acylation. J. Dispers. Sci. Technol..

[36] Buono P., Duval A., Verge P., Averous L., Habibi Y. (2016). New insights on the chemical modification of lignin: Acetylation versus silylation. ACS Sustain. Chem. Eng..

[37] Guo J., Chen X., Wang J., He Y., Xie H., Zheng Q. (2020). The influence of compatibility on the structure and properties of PLA/lignin biocomposites by chemical modification. Polymers.

[38] Vila C., Santos V., Saake B., Parajo J.C. (2016). Manufacture, characterization, and properties of poly-(lactic acid) and its blends with esterified pine lignin. BioResources.

[39] Liang X.L., Liu W.Y., Zhang F.K., Yang W.D., Shi D.J., Chen M.Q. (2019). Effect of maleic anhydride modified lignin on properties of polylactic acid/epoxy soybean oil. Polym. Mater. Sci. Eng..

[40] Guo J.B., Wang J., He Y., Sun H., Chen X.L., Zheng Q., Xie H.B. (2020). Triply biobased thermoplastic composites of polylactide/succinylated lignin/epoxidized soybean oil. Polymers.

[41] Natarelli C.V.L., Lemos A.C.C., de Assis M.R., Tonoli G.H.D., Trugilho P.F., Marconcini J.M., de Oliveira J.E. (2019). Sulfonated kraft lignin addition in urea-formaldehyde resin: Thermokinetic analysis. J. Therm. Anal. Calorim..

[42] Mimini V., Sykacek E., Syed Hashim S.N.A., Holzweber J., Hettegger H., Fackler K., Potthast A., Mundigler N., Rosenau T. (2019). Compatibility of kraft lignin, organosolv lignin and lignosulfonate with PLA in 3D printing. J. Wood Chem. Technol..

[43] Hu J.P., Guo M.H. (2015). Influence of ammonium lignosulphonate on mechanical and thermal properties of polylactic acid/wood fiber biodegradable composites. Acta Mater. Compos. Sin..

[44] Wang N.N., Zhang C.L., Zhu W.Q., Weng Y.X. (2020). Improving interfacial adhesion of PLA/lignin composites by one-step solvent-free modification method. J. Renew. Mater..

[45] Yang Q., Yang J., Gao Z., Li B., Xiong C. (2019). Carbonized cellulose nanofibril/graphene oxide composite aerogels for high-performance supercapacitors. ACS Appl. Energy Mater..

[46] Wang J., Chen X., Zhang C., Akbar A.R., Shi Z., Yang Q., Xiong C. (2019). Transparent konjac glucomannan/cellulose nanofibril composite films with improved mechanical properties and thermal stability. Cellulose.

[47] Tiffany A., Amit R., Cao Y.F., Yuval N., Eldho A., Tal B., Shaul L., Oded S. (2016). Nanocellulose, a tiny fiber with huge applications. Curr. Opin. Biotechnol..

[48] Alana G., Rennan F.S., Derval S. (2020). Nanocellulose from industrial and agricultural waste for further use in PLA composite. J. Polym. Environ..

[49] Huang L.J., Zhang X.X., Xu M.Z., Chen J., Shi Y.H., Huang C.X., Wang S.F., An S.X., Li C.Y. (2018). Preparation and mechanical properties of modified nanocellulose / PLA composites from cassava residue. AIP Adv..

[50] Sullivan E., Moon R., Kalaitzidou K. (2015). Processing and characterization of cellulose nanocrystals/polylactic acid nanocomposite films. Materials.

[51] Zhang Y.C., Cui L., Xu H., Feng X.L., Wang B.J., Pukánszky B., Mao Z.P., Su X.T. (2019). Poly(lactic acid)/cellulose nanocrystal composites via the pickering emulsion approach: Rheological, thermal and mechanical properties. Int. J. Biol. Macromol..

[52] Liu X., Wang W.J., Shao Z.Q., Li L. (2018). Preparation and characterization of nanocellulose/polylactide fully green nanocomposites. Chem. J. Chin. Univ..

[53] Meng L.X., Xu S.Y., Xie Y.Z. (2016). Preparation and characterization of PLA/acetylated nanocellulose films. Sci. Technol. Food Ind..

[54] Xu M.C., Yang R., Huang Q.T., Zhao X., Ma C.H., Li W., Li J., Liu S.X. (2018). Preparation and characterization of acetylated nanocrystalline cellulose-reinforced polylactide highly regular porous films. BioResources.

[55] Wang Z.L. (2014). Preparation and Properties of Nanocellulose/Polylactic Acid Composites. Master’s Thesis.

[56] Bhasney S.M., Bhagabati P., Kumar A., Katiyar V. (2019). Morphology and crystalline characteristics of polylactic acid(PLA)/linear low density polyethylene(LLDPE)/microcrystalline cellulose(MCC) fiber composite. Compos. Sci. Technol..

[57] Fernanda A., Gisele C.V., Maria I.B., Tavares (2017). Effect of microcrystalline and nanocrystals cellulose fillers in materials based on PLA matrix. Polym. Test..

[58] Xian X.J., Wang X.F., Zhu Y.C., Guo Y.T., Tian Y.M. (2018). Effects of MCC content on the structure and performance of PLA/MCC biocomposites. J. Polym. Environ..

[59] Dong F., Yan M.L., Jin C.D., Li S.J. (2017). Characterization of type-II acetylated cellulose nanocrystals with various degree of substitution and its compatibility in PLA films. Polymer.

[60] Li H., Cao Z., Wu D., Tao G., Zhong W., Zhu H., Qiu P., Liu C. (2016). Crystallisation, mechanical properties and rheological behaviour of PLA composites reinforced by surface modified microcrystalline cellulose. Plast. Rubber Compos..

[61] Pei A., Zhou Q., Berglund L.A. (2010). Functionalized cellulose nanocrystals as biobased nucleation agents in poly(*l*-lactide) (PLLA)-crystallization and mechanical property effects. Compos. Sci. Technol..

[62] Saeidlou S., Huneault M.A., Li H.B., Park C.B. (2012). Poly(lactic acid) crystallization. Prog. Polym. Sci..

[63] Zhao B., Zhang Y., Ren H.W. (2020). Effects of microcrystalline cellulose surface modification on the mechanical and thermal properties of polylactic acid composite films. Plast. Rubber Compos..

[64] Frone A.N., Berlioz S., Chailan J.F., Denis M.P. (2013). Morphology and thermal properties of PLA-cellulose nanofibers composites. Carbohydr. Polym..

[65] Song D.L., Zhao Y.L., Dong C.X., Deng Y.L. (2009). Surface modification of cellulose fibers by starch grafting with crosslinkers. Appl. Polym. Sci..

[66] Zhu T., Guo J., Fei B., Feng Z.Y., Gu X.Y. (2020). Preparation of methacrylic acid modified microcrystalline cellulose and their applications in polylactic acid: Flame retardancy, mechanical properties, thermal stability and crystallization behavior. Cellulose.

[67] Hu Y., Tang L., Lu Q., Wang S., Chen X., Huang B. (2014). Preparation of cellulose nanocrystals and carboxylated cellulose nanocrystals from borer powder of bamboo. Cellulose.

[68] Moreno G., Ramirez K., Esquivel M., Jimenez G. (2019). Bio-composite films of polylactic acid reinforced with micro-crystalline cellulose from pineapple leaf fibers. J. Renew. Mater..

[69] Wang H., Pu Y., Ragauskas A., Yang B. (2019). From lignin to valuable products–strategies, challenges, and prospects. Bioresour. Technol..

[70] Thakur V.K., Thakur M.K., Raghavan P., Kessler M.R. (2014). Progress in green polymer composites from lignin for multifunctional applications: A review. ACS Sustain. Chem. Eng..

